# The dual impact of ecology and management on social incentives in marine common-pool resource systems

**DOI:** 10.1098/rsos.170740

**Published:** 2017-08-02

**Authors:** E. S. Klein, M. R. Barbier, J. R. Watson

**Affiliations:** 1 Department of Ecology and Evolutionary Biology, Princeton University, Princeton, NJ, USA; 2 Farallon Institute, Petaluma, CA, USA; 3 Centre for Biodiversity Theory and Modeling, Moulis, France; 4 Stockholm Resilience Centre, Stockholm University, Stockholm, Sweden; 5 College of Earth, Ocean and Atmospheric Sciences, Oregon State University, Corvallis, OR, USA

**Keywords:** common-pool resource, agent-based model, human behaviour, fisheries management, spatial dynamics, cooperation

## Abstract

Understanding how and when cooperative human behaviour forms in common-pool resource systems is critical to illuminating social–ecological systems and designing governance institutions that promote sustainable resource use. Before assessing the full complexity of social dynamics, it is essential to understand, concretely and mechanistically, how resource dynamics and human actions interact to create incentives and pay-offs for social behaviours. Here, we investigated how such incentives for information sharing are affected by spatial dynamics and management in a common-pool resource system. Using interviews with fishermen to inform an agent-based model, we reveal generic mechanisms through which, for a given ecological setting characterized by the spatial dynamics of the resource, the two ‘human factors’ of information sharing and management may heterogeneously impact various members of a group for whom theory would otherwise predict the same strategy. When users can deplete the resource, these interactions are further affected by the management approach. Finally, we discuss the implications of alternative motivations, such as equity among fishermen and consistency of the fleet's output. Our results indicate that resource spatial dynamics, form of management and level of depletion can interact to alter the sociality of people in common-pool resource systems, providing necessary insight for future study of strategic decision processes.

## 1. Introduction

The importance of human behaviour for managing common-pool resources has been recognized for decades (e.g. [1,2]). In marine fisheries, a classic example of a common-pool system [3], insight into human behaviour is critical, especially regarding how people respond to ecological and environmental conditions, as well as management action [4–6]. This is especially important for developing fisheries policies, as management generally acts on people, not fish [7,8].

To better understand human behaviour in common-pool resources, and social–ecological systems more generally, the importance of agent-based modelling has also been recognized for decades (e.g. [9]). Previous applications of agent-based models (ABMs) to fisheries have focused on quantifying the impact of resource variability [10–13], fishing strategies [14,15] and management actions [16–21] on the spatial dynamics of fishermen. However, these studies mostly assess drivers of human behaviour in isolation, while in reality behaviour results from factors acting in concert [1,22–24], and management itself can alter outcomes [1,23,24]. In addition, earlier work exploring differences among individuals has concentrated on intrinsic variability, building in heterogeneity of means, skill or preferences among users [13,14,25–28], as opposed to exploring how heterogeneity itself may arise due to outside influences. Finally, it is important to identify the mechanisms that create behavioural incentives *prior to* exploring how those incentives translate into strategic decisions of resource users (frequently explored with game-theoretic approaches). Often, these are assumed or derived phenomenologically [29], and their identification can be greatly informed by direct interactions with common-pool resource users themselves (e.g. [22]).

Here, we employed informal interviews and an agent-based modelling approach to explore the interaction of management, target species spatial dynamics and resource depletion on the cooperative behaviour of fishermen. We further investigated how external variability due to the stochastic nature of resource exploitation, which interacts with target species ecology and management, can differentiate individuals who would otherwise have the same behavioural incentives. Finally, we assessed how outcomes differed depending on the modelled agent's motivations, whether they sought to maximize their individual catch, equity among fishermen or consistency in the fleet's output through time, as our interviews suggested. This allowed us to evaluate how ecological and management factors shape the *pay-offs* of possible strategies, and how spatial dynamics and management act together as linked mechanisms to create incentives for certain behavioural responses, as opposed to assuming those incentives or strategies *a priori*.

We concentrated on cooperative behaviour through information sharing, as it is central to sustainable collective action and co-management agreements [30,31]. More specifically, we explored the ecological and management conditions that may encourage or deter people from sharing information about the location of a common-pool resource. While the absence of information sharing is not a proof of asocial behaviour (resource users may agree not to interfere with one another, for example), it is one of the most visible forms of cooperation, and therefore often a proxy for collective behaviour more generally. Our focus is on fisheries, and we initiated this work by interviewing individuals within fishing communities on the US West Coast, to provide context and direction for the use of an ABM. With this ABM, we identified the key social and ecological factors affecting information sharing among common-pool resource users.

## 2. Material and methods

### 2.1. Intuition about fishermen's behaviour from interviews

We interviewed fishermen from various sectors in nine fishing communities along the US West Coast. These interviews were informal conversations, without structured questionnaires or surveys. Consequently, they do not enter our research in a quantitative way. Instead, we used them to identify general themes to contextualize our modelling work and to ascertain points of interest in the parameter space of the ABM (for more on the interviews, see electronic supplementary material). The complexity and diversity of social dynamics seen in these qualitative data further stressed the need to first isolate simple external drivers, in particular those specific to resource use, i.e. ecological dynamics and management. In focusing on the human–nature interface, we leave to future work the task of modelling (e.g. with game theory) the complex dynamics that ensue on the human side, involving many other socio-economic factors, to better understand the wide range of behaviours seen in fishing communities.

The first theme identified in the interviews was that the value of information is higher, and therefore sharing more beneficial, for more mobile target species. That is, the variance in spatial dynamics of the target species made a difference in whether fishermen shared information. The second theme was that the way information is shared is strongly influenced by outside drivers, especially those that limit fishing in some way, namely management action and species abundance. For example, for some fishermen, recent shifts from total allowable catch (TAC) limits to individual transferable quotas had increased the potential for open communication. For others, it was cycles of resource abundance and depletion and subsequent changes in market demands that resulted in prosocial behaviour. The third theme was that, while these trends were generally consistent among fishermen within the same fishery, some individuals deviated from the majority. This deviation appeared to arise from intrinsic differences among fishermen, such as in skill and port, but this interacted with how they experienced both external resource uncertainty and management action.

Finally, although much research on common-pool resource users focuses on strict self-interest (e.g. [3]), we found that fishermen's values and motivations could diverge distinctly from such drivers. While making a living was a primary concern for most of the fishermen we spoke with, equity among fishermen, sustainability of the fishery and the resource, and maintaining relationships with others were also significant. In addition, in some cases, landing a consistent harvest over time was more important than maximizing revenue over the season. Of course, these values and motivations are not necessarily at odds with self-interest, and indeed the fishermen we interviewed demonstrated how resource users hold diverse values and experience multiple influences over both the short and long term. However, success is often measured via maximizing total catch or catch rates, and we took this final theme as a reason to explore other measures of success in addition to, but not in lieu of, catch and catch rates.

### 2.2. An agent-based model of fish and fishers

We used an ABM to test the intuition derived from our interviews. We expected that, in certain ecological settings, all fishermen would benefit from the same level of information sharing, broadly correlated to species mobility. In other settings, however, unequal outcomes between fishermen could create diverging incentives; coupled to complex social dynamics, this would produce a variety of outcomes in similar environments. This inequality in outcomes might result only from intrinsic differences between fishermen, e.g. in skill, in which case skilled (successful) and unskilled (unsuccessful) fishermen would have different incentives to share. However, these differences would always exist, no matter how limited the resource was (whether by management limits or by depletion). Thus, we were more interested in unequal outcomes due to resource stochasticity, which could explain the role of catch limits. In that case, incentives differ due to risk-related preferences: risk-seeking agents target the most rewarding behaviours if successful, and risk-averse agents the safest behaviours if unsuccessful.

The purpose of the spatially explicit ABM was to quantify how information-sharing strategies, target species mobility and limitations on catch interact to create this spectrum of success. Thus, the agents were inherently identical, and a model run represented a single fishing season. Additional differences between agents (due to skill or due to economic advantage accrued over multiple seasons) could always be added *a posteriori*, without affecting the basic results of resource and agent spatial dynamics.

This ABM is an extension of one developed in an ecological context to explore the impact of communication between predators on prey consumption rates [32]. It was inspired by mathematical work on search processes, accounting for the trade-off between exploration and exploitation [33]. Here, we expanded this ABM framework to assess a social–ecological common-pool resource system, with fish as prey and fishermen or vessels (hereafter referred to here as ‘fishing agents’) as predators. We further represent management action by adding limits on how much of the resource fishing agents can harvest, and control the renewal and depletion of this resource. A brief description on the major qualitative features of the ABM is provided below (with additional information in the electronic supplementary material), and we refer the reader to [32] for a full description of the model and detailed mathematical analysis.

The ABM is defined on a two-dimensional domain with periodic boundaries (agents crossing a boundary circle over to the other side) that provides a simple and mathematically tractable representation of space that allows us to quantify the trade-offs of exploration and exploitation. This domain is populated with circular fish schools whose radius (*F_s_*) can be changed, affecting the rate at which they are found by fishing agents. All fish schools contain a number of fish, *F_n_*, which controls the reward for finding a school. Finally, the dynamic nature, or mobility, of fish schools sets a limit on how long information about school locations remains valid. To introduce this element of landscape stochasticity while maintaining a constant search difficulty (i.e. a constant number of schools in the domain), schools randomly disappear and are replaced at random locations, following a Poisson process with expected lifespan *τ_l_*.

Fishing agents are also represented simply in the ABM. They can ‘sense’ the local environment in a defined circular region around them. If a fish school is within this sensory zone, they move directly towards the centre of that school, and start harvesting fish at a constant rate, *C_q_*, the ‘catchability’ of the fish. Else, they explore the two-dimensional landscape of the ABM using an intermittent search process, moving ballistically (in a straight line) at a constant speed and pausing at random intervals to pick a new random searching direction. This kind of intermittent search process has been well studied analytically, and is argued in Bénichou *et al.* [33] to capture the most significant features of foraging in a number of natural systems. The expected duration of this process defines the search time for one agent, *τ_s_*, which can be computed explicitly.

When searching on their own, fishing agents choose their random-turn probability to maximize their encounter rate with fish schools. However, this encounter rate is adjusted if agents share information with others. That is, if two agents are modelled to have a social tie, information is shared between them with a probability equal to *λ*, the weight of the tie. These ties are reciprocal, meaning information is shared bi-directionally between the fishing agents. If an agent receives information on a school from another agent via this tie while searching, and there is no alternative fish school near them, they move towards the source of the information. As we are interested in computing the pay-offs for a set level of information sharing, the probability of sharing is not left as a strategic option for agents within the simulation; instead, it is a fixed parameter. Strategic choices are of course important, and we expand on their possible inclusion in our modelling framework, using game-theoretic approaches, for example, in the discussion.

### 2.3. Management and stock depletion

We used this ABM to explore the impact of management limits and resource depletion on the individual catch rates and sociality of fishing agents. Management was implemented in two ways: (i) a TAC and (ii) an individual fishery quota (IFQ). For the TAC scenario, the simulation integration period ended when fishing agents caught a total amount of fish, *T*, collectively, regardless of the catch of each individual fishing agent. This reflects a TAC management limit in the real world, where a fishery closes once that total catch is landed by the fleet as a whole, irrespective of the catch per individual fisherman.

For the IFQ simulations, individual limits (*T_i_*) were set by dividing the TAC by the number of fishing agents. Agents were then removed from the system as they reached their individual quota, *T_i_* = *T*/*N*. This is meant as a simple abstraction of catch share systems, where the catch allowed by management is divided up among the fishermen. In the real world, shares or quotas are assigned to fishermen in more informed ways and are often transferable (i.e. an individual *transferable* quota, ITQ), meaning they can be sold, traded and leased among fishermen. This model does not reflect ITQs, and we did not model these more complicated aspects of catch share or quota systems to maintain as simple a model as possible that can still test the differential impacts of disparate management approaches on agent cooperative behaviour. Furthermore, we do not include assumptions associated with a more strategic modelling approach; therefore, many of the questions around the use of ITQs in reality do not apply here. Instead, our IFQ scenario is meant as a contrasting management approach that allows equal access to the resource among fishing agents versus the TAC.

We ran a second set of simulations where fishing agents can exhaust the resource. Here, instead of a renewable resource drawn from an infinite population (e.g. as in [32]), fishing agents depleted fish schools to progressively lower levels until complete depletion was reached. This was modelled by ensuring that, when a school disappeared, the new school introduced randomly in the system would contain a smaller initial number of fish, proportional to the difference between the total population size *P* and the cumulative harvest of all agents. The parameter *P* represents the total stock and is distinct from the number of fish available in the domain at any given time. In terms of the management scenarios, the allowed level of depletion is given by the fraction *T*/*P*. Given that we are focused on dynamics in a single season, implementing scenarios with and without depletion is realistic, as fisheries in reality may or may not deplete a stock in a single season.

For both management and stock depletion scenarios, simulation outcomes were generated for a wide range of values for fish mobility by modulating the rate at which schools move to random locations (*τ_l_*). We also focused our results on two illustrative cases reflecting species on the US West Coast: a target fish similar to Pacific hake (*Merluccius productus*, hereafter ‘whiting’) and one akin to a demersal species within the groundfish complex (hereafter ‘groundfish’). For the whiting case, the target fish ecology was described with large schools (+*F_s_*), many fish per school (+*F_n_*) and schools that are mobile (−*τ_l_*). By contrast, the groundfish-like species was modelled with smaller schools (−*F_s_*), fewer fish per school (−*F_n_*) and less mobility (+*τ_l_*). As outlined above, the radius of the school, *F_s_*, controls the time it takes for fishing agents to encounter schools, and thus how valuable it is for an agent to receive information about school locations, while the number of fish per school, *F_n_*, and school mobility, *τ_l_*, control the cost of sharing information with others. These exemplar simulation species are not meant to describe the spatial ecology of real species exactly, rather they are used to provide results from two contrasting places in the parameter space of the ABM.

### 2.4. Key control parameters and metrics

In our previous use of this model [32], we focused on fleet-wide adaptation to a target species. Simulations were run until the catch rate *H_i_* for each agent *i* converged to the same asymptotic value *H* (whose analytical derivation is described in the electronic supplementary material). This was used to compute the expected catch per unit of effort (CPUE) under the assumption that effort is proportional to the time spent fishing. It could be optimized by choosing the probability of information sharing, *λ*, uniformly across fishing agents, for a given prey species mobility. Here, we retain this fleet-wide (consensus) level of communication as the parameter representing the strategy of the agents, but now in an environment that includes management limits, and possible depletion of the resource.

In addition, we wished to address the variety of preferences that may drive resource users to adopt social behaviour, and there are different objectives towards which one could adjust the sharing level, *λ*, of fishing agents in the ABM. Therefore, we also did not compute its optimal or evolutionary value for one objective, but instead systematically explore different metrics of success over the whole range of *λ*. All those metrics are constructed from dynamically measuring *H_i_* (*λ*,*t*), the catch rate of agent *i* at time *t* for a given *λ*.

Of those possible, the metric we focused on was the catch rate averaged both across fishing agents and over the simulation period, *H*. We also compared this with two other observables reflecting additional motivations outlined by fishermen in our interviews. First, the variance in CPUE between agents, averaged over time, which denotes inequity among fishermen in the fleet. The second is the variance over time, averaged across agents, which represents the consistency of the fleet's catch during a fishing season, or steadiness of the ‘resource flux’ from fishermen to buyers. These three objectives are defined as follows:

2.1 $$\begin{array}{rl} O_{\mathrm{CPUE}} & =\frac{{\mathrm{avg}}_{i}\;{\mathrm{avg}}_{t}\;H_{i}(\lambda ,t)}{\max_{\lambda }\;{\mathrm{avg}}_{i}\;{\mathrm{avg}}_{t}\;H_{i}(\lambda ,t)}, \end{array}$$

2.2 $$\begin{array}{rl} O_{\mathrm{equity}} & ={\left(\frac{{\mathrm{std}}_{i}\;{\mathrm{avg}}_{t}\;H_{i}(\lambda ,t)}{\min_{\lambda }\;{\mathrm{std}}_{i}\;{\mathrm{avg}}_{t}\;H_{i}(\lambda ,t)}\right)}^{-1} \end{array}$$

2.3 $$\begin{array}{rl} \mathrm{and}\;\;O_{\mathrm{stability}} & ={\left(\frac{{\mathrm{std}}_{t}\;{\mathrm{avg}}_{i}\;H_{i}(\lambda ,t)}{\min_{\lambda }\;{\mathrm{std}}_{t}\;{\mathrm{avg}}_{i}\;H_{i}(\lambda ,t)}\right)}^{-1}, \end{array}$$

where avg*_x_ f*(*x,y*) and std*_x_ f*(*x*,*y*) denote, respectively, the average and standard deviation of some function *f*(*x*,*y*) over variable *x*. They are constructed so that *O*_CPUE_ increases with the collective expected catch rate, *O*_equity_ decreases with variance between agents and *O*_stability_ decreases with variance in landed catch over time. All three are normalized to facilitate comparisons across different settings: for any ecological situation, they will be equal to 1 at the optimal level of information sharing, and smaller for suboptimal strategies. These metrics are reflective of some motivations reported by the fishermen we interviewed (electronic supplementary material). For example, maximizing *O*_equity_ would occur to socially minded fishermen who may aim for equity among the fleet, and maximizing *O*_stability_ is indicative of fishermen who look to land a consistent harvest. Together, these three objectives, catch rate, equity and flux stability, represent a first step towards exploring the wide range of motivations behind harvester behaviour.

## 3. Results

### 3.1. Exploring the effects of management

Our simulations of management action alone limited the amount of fish agents could catch, by either a TAC or IFQ, while the density of fish in the system remained constant (but harder to exploit if more mobile). The introduction of either limit created variance among agents' CPUE within the modelled fleet. Let us recall that we chose to model agents as identical: as we argued before, if they differed in skill, their intrinsic variance in CPUE would not depend on catch limits. On the other hand, extrinsic variance due to stochasticity is sensitive to management.

To understand this extrinsic variance, fishing can be thought of as a gambling process, with fishing agents more or less successful depending on how often they encounter the randomly distributed and moving schools. Each successful or failed encounter to find a school can be thought of as a trial. The more trials there are, the lower the variance between agents at the end of the run, as many random catches average out, but this depends on both management limits and the spatial dynamics of the target. For example, a shorter season means fewer trials and hence more variance. In addition, if the target species gathers in either very mobile or very small schools, then there are more trials and less variance. In both cases, more encounters must occur before the allowed amount is harvested, because schools either move away quickly or contribute little per encounter.

**Figure 1.**
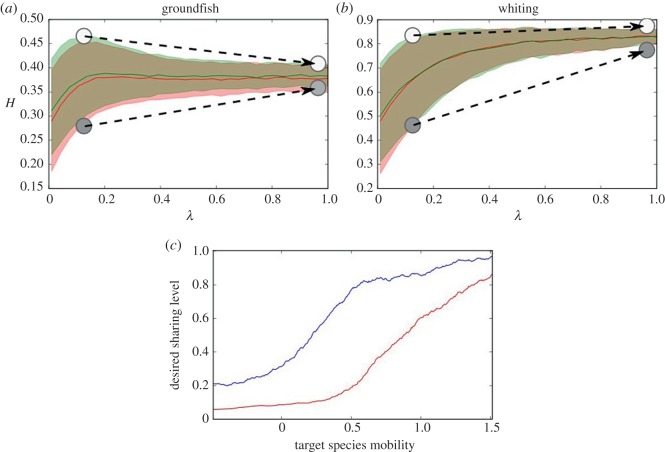
Differential impacts of sharing on fishing agents. (*a,b*) The spread of fisherman success as a function of information sharing under a finite harvest limit, with the average harvest rate for the whole fleet (solid line) and 80% interval (filled area), under a TAC (red) or IFQ (green) for groundfish (*a*) and for whiting (*b*). Note that the red and green areas overlap, demonstrating that outcomes of either management approach are the same in terms of catch per unit of effort (CPUE), *H*. The dots and arrows indicate the change in CPUE for ‘successful’ (white dots) and ‘unsuccessful’ (grey dots) fishing agents as sharing increases. Panel (*c*) reveals the level of information sharing (*y*-axis) that allows agents to optimize catch per unit of effort (CPUE) across the range of target species mobility (*x*-axis) for ‘successful’ (red, 90th percentile) and ‘unsuccessful’ (blue, 10th percentile) fishing agents.

In figure 1*a,b*, across a range of information sharing (*λ* = 0.0–1.0, *x*-axis), the fleet-wide average CPUE is shown by the solid lines, red for TACs and green for IFQ, while the similarly coloured shaded areas denote the variance in individual CPUE between the least and most successful agents (note these shaded areas overlap in figure 1). For both case studies, groundfish and whiting (figure 1*a,b,* respectively), this variance decreases as information sharing increases, as sharing means multiple agents have the same information and harvest the same schools. There remains some variance even with full information sharing (*λ* = 1), because the initial discoverer of a school retains a moderate advantage by having additional time to exploit it before others arrive.

Yet, for certain target species, fishing agents do not benefit equally from communication, depending on their success during the season. Although the identity of successful agents changes from season to season, knowing how much variance exists between agents in one season is important for exploring a variety of objectives (such as social inequalities and risk aversion). For example, in figure 1*a,b*, white dots represent a successful agent, that is an agent who is ‘lucky’ and more often finds schools, and the grey an unsuccessful one (again, apart from this ‘luck’ in being near schools, agents are identical and there is not inherent ability or success). As sharing increases along the *x*-axis, the arrows in figure 1*a,b* highlight the change in CPUE for a successful and an unsuccessful agent given that amount of sharing in the model run. For the whiting case, both unsuccessful and successful agents increase CPUE in model runs with sharing, with agents who are unsuccessful more than doubling their harvest rate. For groundfish, while an agent who is generally unsuccessful can increase its CPUE by 50% with information sharing, the successful agent sees a 15% decline in CPUE compared to what it would achieve optimally in the scenario with low sharing (*λ* = 0.1). We recall that sharing is not changed during a model run: our goal is to compute the range of pay-offs associated with different levels of sharing, each tested in a separate model run. Further, sharing is an open process: a successful agent cannot ‘hide’ from an unsuccessful agent requesting information.

Figure 1*c* highlights this effect across a range of target species mobility (*x*-axis). On the *y*-axis, we indicate the sharing level *λ* that would maximize CPUE, and plot this for the most successful and unsuccessful agents—hereafter defined as the top and bottom 10% of agents in terms of CPUE (red and blue lines, respectively). Information sharing is always more favourable for unsuccessful than successful agents, but the divergence between their optima depends on target species dynamics. Across species mobility, this difference is greatest for a mid-range target, and only minimized in extreme cases of very high- or low-mobility target species, where sharing, respectively, benefits or hinders everyone more equally.

If some or all of the difference between successful and unsuccessful agents came from their individual skill at finding schools, many of the same intuitions would hold: variance would still be reduced by sharing, and the largest divergence between optimal levels of information sharing would still be found at intermediate mobility. However, this heterogeneity would not depend on resource stochasticity amplified by catch limits, and therefore, it would be insensitive to management action.

Perhaps surprisingly, CPUE variance between agents happens to be computationally identical for the TAC and IFQ simulations (figure 1*a,b*, the red and green shades overlap). While this exact equality is a peculiarity of the model, it allows us to point to an important intuition, as these two variances result from different mechanisms. Under a TAC, the variation among agents lies in what fraction of the total each fishing agent is able to procure before the end of the simulation period. Under IFQs, this variation is due to the time it takes to harvest their allotment. In other words, the variance among agents in the TAC system revolves around a benefit, individual total catch, whereas for the IFQ system it revolves around a cost, the time (or effort) spent fishing. One will be more favourable than the other if we do not put the same weight on benefits and costs.

There is a further difference between management types: under an IFQ system, unsuccessful agents remain active longer, while others reach their quotas and stop fishing, meaning they have access to less and less information about the location of fish schools, further lowering their CPUE. As we now discuss, this effect is amplified by depletion.

### 3.2. The impact of stock depletion

In the stock depletion simulation experiments, both the total catch allowed by management limits *T* and the fish population *P* are finite. Depletion is implemented by progressively limiting fish renewal, thereby allowing agents to increasingly reduce the resource, which translates into more time spent searching per unit of fish harvested.

**Figure 2.**
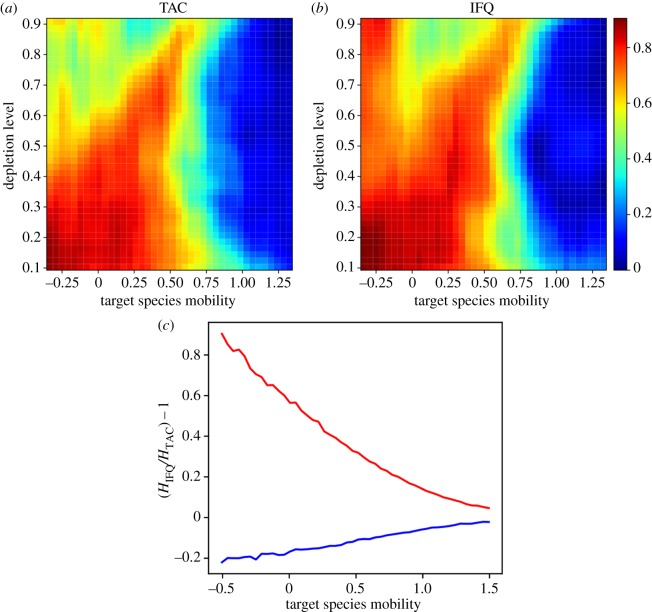
Divergence of optimal information-sharing level among fishing agents across species mobility (*x*-axis). The top two panels show how this changes with depletion (*y*-axis) depending on management action: TAC (*a*) and IFQ (*b*). This divergence is represented by 1 − (*λ*+/*λ*−) as a function of species mobility (on a log scale) and depletion level, where *λ*+ is the optimal sharing level for ‘successful’ fishermen, and *λ*− is optimal for ‘unsuccessful’ fishermen (lowest 10%). When this value is close to 0 (blue), there is no divergence and all agents can optimize their CPUE by a similar level of sharing, whereas values closer to 1 represent increasing divergence (warmer colours). (*c*) compares TAC and IFQ at high depletion: average value of $(H_{\mathrm{IFC}}/H_{\mathrm{TAC}})-1$ for successful (red) and unsuccessful (blue) fishing agents, demonstrating that ‘unsuccessful’ fishermen exhibit better CPUE under a TAC, whereas ‘successful’ fishermen do better under an IFQ system.

The effects of depletion are highlighted in figure 2. In figure 2*a,b*, colours indicate the difference between the optimal levels of information sharing (i.e. *λ* that maximizes CPUE) for successful and unsuccessful agents, for the TAC (figure 2*a*) and IFQ (figure 2*b*) scenarios. Cooler colours indicate little difference between agents; in this case, neither benefit from sharing. Warmer colours denote a difference in the need to share to maximize CPUE between successful and unsuccessful agents, with the latter benefiting from a higher level of information sharing. Note that, as before, there is a greater disparity in the usefulness of sharing for low (e.g. our groundfish example) and mid-range mobility species (red on the left side of the panel in figure 2*a,b*). Depletion results in two new patterns in this parameter space. The first is that, as depletion increases (*y*-axis in figure 2*a,b*), disparity decreases among agents on the importance of sharing, especially for high- and low-mobility species (the decrease of warm colours moving up the *y*-axis), e.g. our whiting and groundfish cases, respectively. That is, depletion means agents are more likely to agree not to share, as depletion reduces the benefit of doing so relative to its cost when resources become scarce. The second pattern reveals a difference between the TAC and IFQ scenarios. Under IFQs, for low-mobility species such as groundfish and at high levels of depletion, there is a greater difference in the desired level of sharing than there is under TACs (difference in warm colours in the top left corner of figure 2*a,b*). For IFQs alone, unsuccessful fishing agents in that ecological setting benefit far more from information sharing than successful agents.

This differential impact of IFQs is consequential for the behaviour and success of fishing agents. Figure 2*c* shows the ratio of optimal CPUEs under TACs and IFQs ($(H_{\mathrm{IFC}}/H_{\mathrm{TAC}})-1$, *y*-axis) at high depletion (*T*/*P* = 0.9) for successful (red line) and unsuccessful (blue line) fishing agents, over a range of species mobility. Here, positive numbers denote better catch rates, *H*, under IFQs, and negative numbers better rates under a TAC. The most striking result is that, for all conditions, an IFQ management system provides improved CPUE for successful fishing agents (figure 2*c*, red line), and, conversely, TACs are better for unsuccessful fishing agents (figure 2*c*, blue line). This pattern emerges because, in the TAC scenario, all agents remain in the model for the duration of the simulation and thus all encounter the effects of depletion equally, with fish increasingly difficult to find. In the IFQ simulations, more successful fishermen acquire their quota more quickly, and consequently, are able to leave the fishery earlier, when depletion is less pronounced. By contrast, unsuccessful fishermen experience depleted conditions for longer periods of time in the IFQ scenario, with fish schools becoming increasingly difficult to find.

### 3.3. Alternative measures of success

**Figure 3.**
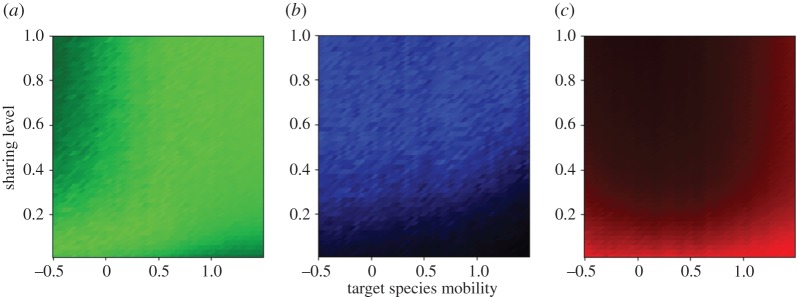
The range of possible preferences fishing agents can optimize by their level of information sharing, given species mobility (at low depletion, under a TAC). For all panels, the colour at each point of the parameter space is determined by one of the three objective functions. In (*a*), this is *O*_CPUE_ (green), in (*b*) it is *O*_equity_ (blue) and in (*c*) it is *O*_stability_ (red). Brightness of hue (luminosity) indicates areas where that objective function is maximized across target species mobility (*x*-axis) and sharing level (*y*-axis). For example, increased sharing for fishing agents harvesting moderately to highly mobile fish schools in our model could optimize both catch per unit of effort, CPUE (*a*, *O*_CPUE_), and equity among agents (*b*, *O*_equity_), but may end up with inconsistent catches (absence of colour in *c*, *O*_stability_).

So far, we have shown how different agents within the fleet may see their time-averaged CPUE affected by sharing strategies, management limitations and resource depletion. Yet our US West Coast interviews suggested that fishermen can measure their success in a variety of ways, for example in terms of income equity across the whole fleet, represented here by the objective function *O*_equity_ (equation (2.2)), or the consistency of collective harvest, measured by *O*_stability_ (equation (2.3)). Figure 3 shows how well fleets perform according to these metrics, given their level of information sharing (*y*-axis) across the range of species mobility (*x*-axis). The luminosity of colours reflects how close each success metric is to its maximum: in the RGB representation, the green is *O*_CPUE_ (figure 3*a*), the blue is *O*_equity_ (*b*), and the red, *O*_stability_ (*c*). For the additional two objectives, optimal behaviour is easily understood: in any setting and for all agents, information sharing always enhances equity (increase in blue up the *y*-axis in figure 3*b*), and detracts from flux stability (absence of red hue beyond very low sharing values on the *y*-axis in *c*). Thus, we see that collective catch rate maximization is incompatible with prosociality for resident species, while it is at odds with flux stability for intermediate and mobile species.

## 4. Discussion

Previous work [32] found that certain ecological factors, especially target species mobility, controls cooperation in the form of information sharing between fishing agents. However, complex social dynamics could take place if information sharing is not equally attractive to all agents. This arises naturally when agents are not equally successful. If the difference is due to skill, then species mobility is the only factor, among those considered here, that will decide whether skilled and unskilled agents agree on how much information to share. This does not provide an explanation for how, for instance, management action can create new dilemmas.

Even if agents are equal in skill, inequalities within a fishing season arise from chance, and agents can differ in risk preference: different information-sharing strategies can be adopted to attain maximal expected pay-off, or minimal risk. In this case, the results of our ABM identify that harvest limits from management action lead to increased variance in terms of catch per unit effort (CPUE) during one season. Consequently, individuals within the same fleet, targeting the same species under the same management strategy will not benefit equally from information sharing or changes in management. This disparity is a direct result of the interaction between the spatial dynamics of the resource and a constraint that disrupts harvesting. While revealed by our specific simulation model, this mechanism is in fact very general: all factors limiting the fishing volume or length of the simulation increase the inherent stochasticity associated with catching fish, and cause the benefits of collective action to be unequal, even within a group of identical agents.

In terms of social behaviour, this variance has a differential effect on the value of information between unsuccessful and successful fishing agents (figure 1), whose success is due to their ‘luck’ in being near randomly occurring fish and not inherently modelled differences. This suggests that a harvest limit can cause risk-averse fishermen (wanting to minimize potential losses) and risk-seeking ones (wanting to maximize potential gains) to benefit, respectively, from more and less information sharing than is optimal for the whole fleet. This dilemma is important for real-world fisheries, as it indicates the possibility for suboptimal collective decision-making, tensions within a given fleet and incentives for spying behaviour. While not yet employing assumptions of strategic decision-making among the agents, the mechanism proposed here exposes the relationship between these dilemmas and prey ecology.

Furthermore, this work shows that diverging social incentives may result from the interaction of these outside drivers alone. Other models (e.g. [13,14,25–27]) have considered the possibility of agents being intrinsically heterogeneous, to reflect skill or access to additional resources (gear, technology or capital). However, those intrinsic differences are independent of target species ecology or management scheme, and cannot mediate their influence on social behaviour. Here, we investigate a distinct and previously unexplored phenomenon, where the interaction of management, resource depletion and stochastic dynamics of the resource can create diverging incentives for information sharing (i.e. sharing information is more or less beneficial) where there were none before.

The divergence found here depends on target species mobility. For our case studies, agents in the groundfish example exhibited more heterogeneous benefits from sharing than for whiting (Pacific hake, *Merluccius productus*; figure 1*a,b*). This difference was greatest for target species in the mid-range of mobility (figure 1*c*). These insights allow us to make inferences even about behaviour that was not explicitly modelled, such as spying. We would expect spying to occur mostly among common-pool users when resources meet three criteria: (i) high uncertainty that results in the information held by others being valuable; (ii) sufficiently large and slow-moving schools that a spying user benefits from them before they relocate or are entirely harvested by the finder; (iii) not large and mobile enough that the finder cannot exhaust them alone (therefore removing any cost to reciprocal sharing).

Our findings also reveal potentially unanticipated differences in how fishermen will react to catch share systems. Individual quotas, or catch shares, are often advocated over TACs because they are expected to increase economic efficiency, decrease overcapitalization and aid in sustainability [34–36]. We found that, in the absence of resource depletion, neither unsuccessful nor successful fishing agents did better in terms of CPUE when the management approach was shifted from a TAC to an IFQ. However, the variance in catch rate among the agents with either management strategy has different origins. Under a TAC, it concerns the share of catch, a benefit, and under an IFQ, the time spent fishing, a cost. This indicates that the form of management has the potential to incentivize users towards different objectives, and outcomes may vary if fishermen prefer to optimize benefits (e.g. value of catch) or minimize costs (e.g. fuel or risk). It is important to note that these outcomes are only equivalent if CPUE, the cost–benefit ratio, is the measure of success.

We also uncovered a difference among agents’ success depending on management approaches when depletion was added to our simulations, indicating differing responses among fishermen to management action when fishing depletes a stock. When such resource depletion was significant in our model, IFQs generally benefited fishing agents who were most successful, whereas agents who were less so did better under TACs. This is due to the increased difficulty of finding fish as the season advances: it strongly disadvantages unsuccessful agents under an IFQ system, as they take more time to acquire their quota (i.e. increasing costs). This effect is compounded with the decreasing number of partners with whom to share information, and is exacerbated when the resource is less mobile.

This result supports the theory that catch share systems, which we represented with IFQs, may encourage less successful fishermen to leave the fishery, increasing overall economic efficiency but also consolidating access. However, our findings reveal a novel source of these dynamics, and indicate they can emerge in a single season due to the interaction of resource dynamics, management and social networks. In addition, catch shares are expected to act as incentives and not harvest controls, and to encourage cooperation and reduce competition [33]. Our results suggest that fishermen may not all experience them as incentives, even within a single fleet, and that a shift from a TAC to shares may elicit contrasted reactions (figure 2). Finally, divergent incentives to share information suggests increased potential for spying and competition, especially under certain ecological conditions, not cooperation or open communication as may be expected under catch shares [34,37].

Further effects arise if that resource can be depleted locally or seasonally. While we do not model successive seasons, or the potential for quotas to extend the season and reduce the race to fish due to secured resource rights, the simulations where fishing agents deplete the resources mirrors a situation where management limits are unable to avoid either seasonal or local resource exhaustion. As Huang & Smith [38] note, this is a concern for the implementation of catch shares. Our results support their conclusion that management needs to address the timing of exploitation within a season if catch shares are to have desired outcomes. Given that our results indicate that the differences in success among agents under our ITQs are related to the cost of taking longer to find fish, this may be of increased concern in fisheries where spending more time searching incurs greater consequences, for example in terms of price and use of fuel or risks to the fishermen themselves.

It is critical to note that our approach does not make assumptions about strategic decisions of the resource users, i.e. we do not invoke a game-theoretic approach or aim to understand how outcomes would impact strategic behaviour or the resource users. For example, agents do not ‘choose’ whether or not to share information, but instead we simply address how sharing would be more or less valuable. Including such decision processes would require additional assumptions and model complexity, especially as our interviews suggest a large range of socio-economic factors at play. The added complexity of considering social dynamics is twofold. First, combining strategic interactions with spatial dynamics makes the spectrum of possible behaviours so vast that it cannot be explored exhaustively. Instead, we can only formulate some heuristics, inspired by models of human cognition, and compare them, e.g. in a tournament setting (e.g. [39]). Second, the social consequences of these strategies must also be considered. For instance, fishermen who belong to the same off-water community may be more reluctant to employ antagonistic strategies, which would prove damaging to other facets of their livelihood.

Instead, we focus on limiting model assumptions and intricacy in the interest of more clearly understanding the mechanism through which resource variability and spatial dynamics impact social resource users under management and resource depletion. Future research is encouraged that engages our findings in a game-theoretic arena. Another clear way forward is to find empirical evidence of these mechanisms in real-world fisheries beyond our interviews. Doing so as well as expanding our work to allow assumptions of strategic behaviour are both essential next steps.

Finally, our results focused mainly on time-averaged CPUE as the metric of fishing agent success, but in reality, resource users do not always have the desire or means to optimize this cost–benefit ratio. For example, fishermen may want to avoid inequities or competitive behaviours like spying, which can be a source of stress and conflict. They can do so by reducing the stochastic variance between fishermen in CPUE that creates diverging incentives, in particular by sharing more information than is optimal for CPUE. From our interviews, we also learned that fish processing companies can play a central role in fisheries and ports, controlling fishermen's behaviour to obtain landings in a steady flux. This can be achieved by a lower level of information sharing than would optimize CPUE, which reduces correlation between fishermen and allows a smoother collective output, akin to a portfolio effect in finance. Figure 3 indicates that such focus on stability reduces the ability of agents to maximize CPUE or equity – and this was indeed reflected in our interviews with fishermen (electronic supplementary material). Overall, our final results in figure 3 indicate the range of behaviour beyond CPUE optimization that is possible, a range echoed by the fishermen themselves (electronic supplementary material). These findings demonstrate how the model can capture a diversity of motivations, even without structural changes in the simulated agents. Accompanying an exploration of these modelling possibilities with social science research regarding real-world motivations would help focus that exploration on useful areas of the parameter space. In this way, simulation results could be used to construct hypotheses about the underlying objectives of fishermen in real-world marine systems, and provide an avenue for testing those hypotheses with empirical evidence. Such cross-disciplinary endeavours would reflect a greater range of motivations and behaviour beyond cost–benefit optimization, and advance our understanding of the role of human behaviour in social–ecological systems.

## 5. Conclusion

One piece of the puzzle of natural resource use is the interface between ecological and human factors. By focusing on that piece, we propose to understand mechanistically how spatial resource dynamics and management action create a set of incentives and pay-offs for cooperative behaviour, which then play into a more complex socio-economic picture. We find the interaction of fishery limits, social behaviour and target species ecology can impact individuals differently both within and across groups harvesting a common-pool resource, and identify potential conditions under which we may expect heterogeneous cooperative behaviour in users who would otherwise behave the same. Our results also suggest local depletion and management approaches can influence the collectively optimal level of cooperation, and cause users to respond unexpectedly to management changes beneficial in other sectors and for other people. These influences can initiate emergent differences among individuals, based on their success or risk aversion, that may manifest as diverging incentives to cooperate or differing responses to management, even as they would perform identically in the absence of management limits or resource depletion. While results are based on a simplified model of what is a very complex system, the qualitative mechanisms they reveal are intuitive and robust, hinging on the intrinsic uncertainty of fishing, and we see preliminary supporting evidence in our interviews.

Understanding when the information held by others is valuable, how it is transferred, and the resulting behaviours and relationships is critical for managing common-pool resources like fisheries. While our results provide initial indications for how target species ecology and management can influence human behaviour, our model as well as our interviews with members of fishing communities suggest the importance of additional factors. In reality, pressures acting on people are complex and result in diverse outcomes, even within groups targeting the same resource [1,40]. These motivations and behaviours are not limited to typical measures of success, and they cannot be abstracted as simply as we do so here. However, our results strongly indicate the need to address both ecological and human dynamics in planning management action, and for further work and collaboration on understanding human behaviour in common-pool resource systems.

## Supplementary Material

Supplementary Information

## Data accessibility

No data were used in this modelling research. The ABM is provided in [32].

## References

[1] Hilborn R 1985 Fleet dynamics and individual variation: why some people catch more fish than others. Can. J. Fish. Aquat. Sci. 42, 2–13. (doi:10.1139/f85-001)

[2] Ostrom E, Burger J, Field CB, Norgaard RB, Policansky D 1999 Revisiting the commons: local lessons, global challenges. Science 284, 278–282. (doi:10.1126/science.284.5412.278) 10195886 10.1126/science.284.5412.278

[3] Hardin G 1968 The tragedy of the commons. Science 162, 1243–1248. (doi:10.1126/science.162.3859.1243) 5699198

[4] Wilen JE, Smith MD, Lockwood D, Botsford LW 2002 Avoiding surprises: incorporating fisherman behavior into management models. Bull. Mar. Sci. 70, 553–575.

[5] Levin SA, Lubchenco J 2008 Resilience, robustness, and marine ecosystem-based management. Bioscience 58, 27–32. (doi:10.1641/b580107)

[6] Fulton EA, Smith ADM, Smith DC, van Putten IE 2011 Human behaviour: the key source of uncertainty in fisheries management. Fish Fish. 12, 2–17. (doi:10.1111/j.1467-2979.2010.00371.x)

[7] Jentoft S 1997 Five truisms of fisheries management In Paper presented at the Vilamoura International Meeting on Fisheries: Multiple Objectives and Fisheries Management—Strategies for the Future, 3–4 November 1997, Vilamoura, Portugal.

[8] Wilson D, McCay B. 2001 Fishery management, human dimension In Marine policy and economics: a derivative of the encyclopedia of ocean sciences (eds Steele JH, Thorpe SA, Turekian KK, pp. 1023–1028. Burlington, MA: Academic Press.

[9] Deadman PJ 1999 Modelling individual behaviour and group performance in an intelligent agent-based simulation of the tragedy of the commons. J. Environ. Manage. 56, 159–172. (doi:10.1006/jema.1999.0272)

[10] Bousquet F, Cambier C, Mullon C, Morand P, Quensiere J 1994 Simulating fishermen societies. In Simulating societies (eds Gilbert N, Doran J), pp. 143–164. London, UK: UCL Press.

[11] Dreyfus-Leon M, Gaertner D 2006 Modeling performance and information exchange between fishing vessels with artificial neural networks. Ecol. Model. 195, 30–36. (doi:10.1016/j.ecolmodel.2005.11.006)

[12] Millischer L, Gascuel D 2006 Information transfer, behavior of vessels and fishing efficiency: an individual-based simulation approach. Aquat. Living Resour. 19, 1–13. (doi:10.1051/alr.2006001)

[13] Little LR, McDonald AD 2007 Simulations of agents in social networks harvesting a resource. Ecol. Model. 204, 379–386. (doi:10.1016/j.ecolmodel.2007.01.013)

[14] Brede M, De Vries HJM 2010 Harvesting heterogeneous renewable resources: uncoordinated, selfish, team-, and community-oriented strategies. Environ. Modell. Softw. 25, 117–128. (doi:10.1016/j.envsoft.2009.07.007)

[15] Hicks RL, Horrace WC, Schnier KE 2012 Strategic substitutes or complements? The game of where to fish. J. Econom. 168, 70–80. (doi:10.1016/j.jeconom.2011.09.007)

[16] Elliston L, Cao L 2006 An agent-based bioeconomic model of a fishery with input controls. Math. Comput. Model. 44, 565–575. (doi:10.1016/j.mcm.2006.01.010)

[17] Moustakas A, Silvert W, Dimitromanolakis A 2006 A spatially explicit learning model of migratory fish and fishers for evaluating closed areas. Ecol. Model. 192, 245–258. (doi:10.1016/j.ecolmodel.2005.07.007)

[18] Soulié J.-C, Thébaud O 2006 Modeling fleet response in regulated fisheries: an agent-based approach. Math. Comput. Model. 44, 553–564. (doi:10.1016/j.mcm.2005.02.011)

[19] Vermard Y, Marchal P, Mahevas S, Thebaud O 2008 A dynamic model of the Bay of Biscay pelagic fleet simulating fishing trip choice: the response to the closure of the European anchovy (*Engraulis encrasicolus*) fishery in 2005. Can. J. Fish. Aquat. Sci. 65, 2444–2453. (doi:10.1139/f08-147)

[20] Little LR, Punt AE, Mapstone BD, Begg GA, Goldman B, Williams AJ 2009 An agent-based model for simulating trading of multi-species fisheries quota. Ecol. Model. 220, 3404–3412. (doi:10.1016/j.ecolmodel.2009.08.004)

[21] Toft JE, Punt AE, Little LR 2011 Modelling the economic and ecological impacts of the transition to individual transferable quotas in the multispecies US west coast groundfish trawl fleet. ICES J. Mar. Sci. 68, 1566–1579. (doi:10.1093/icesjms/fsr095)

[22] Holland DS, Sutinen JG 2000 Location choice in New England trawl fisheries: Old habits die hard. Land Econ. 76, 133–150. (doi:10.2307/3147262)

[23] Salas S, Gaertner D 2004 The behavioural dynamics of fishers: management implications. Fish Fish. 5, 153–167. (doi:10.1111/j.1467-2979.2004.00146.x)

[24] Branch TA et al. 2006 Fleet dynamics and fishermen behavior: lessons for fisheries managers. Can. J. Fish. Aquat. Sci. 63, 1647–1668. (doi:10.1139/f06-072)

[25] Allen PM, McGlade JM 1986 Dynamics of discovery and exploitation—the case of the Scotin Shelf groundfish fisheries. Can. J. Fish. Aquat. Sci. 43, 1187–1200. (doi:10.1139/f86-148)

[26] Brede M, Boschetti F, McDonald D 2008 Strategies for resource exploitation. Ecol. Complex. 5, 22–29. (doi:10.1016/j.ecocom.2007.07.002)

[27] Cabral RB, Geronimo RC, Lim MT, Alino PM 2010 Effect of variable fishing strategy on fisheries under changing effort and pressure: an agent-based model application. Ecol. Model. 221, 362–369. (doi:10.1016/j.ecolmodel.2009.09.019)

[28] Grainger CA, Costello C 2015 Distributional effects of the transition to property rights for a common-pool resource. Mar. Resour. Econ. 31, 1–26. (doi:10.1086/684132)

[29] Fenichel EP, Abbott JK, Huang B 2013 Modelling angler behaviour as a part of the management system: synthesizing a multi-disciplinary literature. Fish Fish 14, 137–157.

[30] Walker JM, Gardner R, Ostrom E 1990 Rent dissipation in a limited-access common-pool resource: experimental evidence. J. Environ. Econ. Manage. 19, 203–211. (doi:10.1016/0095-0696(90)90069-B)

[31] Ostrom E, Walker J, Gardner R 1992 Covenants with and without a sword: self-governance is possible. Am. Polit. Sci. Rev. 86, 404–417. (doi:10.2307/1964229)

[32] Barbier M, Watson JR 2016 The spatial dynamics of predators and the benefits and costs of sharing information. PLoS Comput. Biol. 12, e1005147 (doi:10.1371/journal.pcbi.1005147) 27764098 10.1371/journal.pcbi.1005147 PMC5072596

[33] Bénichou O, Loverdo C, Moreau M, Voituriez R. 2011 Intermittent search strategies. Rev. Mod. Phys. 83, 81–129 (doi:10.1103/RevModPhys.83.81) 10.1103/PhysRevE.80.031146 19905101

[34] Grafton RQ 1996 Individual transferable quotas: theory and practice. Rev. Fish Biol. Fisher. 6, 5–20. (doi:10.1007/bf00058517)

[35] Costello C, Gaines SD, Lynham J 2008 Can catch shares prevent fisheries collapse? Science 321, 1678–1681. (doi:10.1126/science.1159478) 18801999 10.1126/science.1159478

[36] Branch TA 2009 How do individual transferable quotas affect marine ecosystems? Fish Fish. 10, 39–57. (doi:10.1111/j.1467-2979.2008.00294.x)

[37] Griffith DR 2008 The ecological implications of individual fishing quotas and harvest cooperatives. Front. Ecol. Environ. 6, 191–198. (doi:10.1890/050060)

[38] Huang L, Smith MD 2014 The dynamic efficiency costs of common-pool resource exploitation. Am. Econ. Rev. 104, 4071–4103. (doi:10.1257/aer.104.12.4071)

[39] Rendell L et al. 2010 Why copy others? Insights from the social learning strategies tournament. Science 328, 208–213. (doi:10.1126/science.1184719) 20378813 10.1126/science.1184719 PMC2989663

[40] Palmer CT 1991 Kin selection, reciprocal altruism, and information sharing among Maine lobstermen. Ethol. Sociobiol. 12, 221–235. (doi:10.1016/0162-3095(91)90005-b)

