# Supplementary material for: The dual impact of ecology and management on social incentives in marine common-pool resource systems
Source: R Soc Open Sci. 2017 Aug 2;4(8):170740. doi: 10.1098/rsos.170740 (PMC5579129; doi:10.1098/rsos.170740)
Supplement: Supplementary Information [file rsos170740supp1.docx]

Klein ES, Barbier MR, Watson JR.: “The dual impact of ecology and management on social incentives in marine common pool resource systems.”

## SUPPLEMENTARY INFORMATION

### Interviews with Oregon and California fishing community

We conducted semi-structured interviews with nine fisheries communities along the West coast (Oregon and California), engaging fishermen across various sectors, as well as academics, managers, fisheries scientists, industry and processing representatives, non-governmental organizations, and debriefers and observers from the NOAA West Coast Groundfish Observing program (Table 1, May 2014 and January 2015; Princeton IRB Protocol #0000006705). These interviews were informal conversations, without structured questionnaires or surveys, and therefore were used to set the context of our modeling work and identify points of interest in the parameter space of the ABM. Interviews began with a set of open-ended questions about the general structure of social networks and the potential and motivations for sharing or other social behavior within the fishery and the local fleet, as well as further motivations or limitations on harvesting itself. Discussion was allowed to progress naturally from there. Interviews lasted for one to several hours, depending on the interest and availability of the interviewee.

The value of information and the need for sharing among fishermen targeting highly mobile species (e.g. whiting or Pacific hake, *Merluccius productus*, and pink shrimp, *Pandalus jordani*) was reported consistently, as was the converse independence of groundfishermen targeting more sedentary species within the groundfish complex. Influences acting on the sharing of information were also relatively consistent among fishermen within the same fishery. These included outside drivers that limited fishing in some way, namely management and species abundance. For example, many whiting fishermen noted that a shift in management approach from total allowable catch limits to individual transferrable quotas had increased the potential for open communication. Previously, the fishery had been more competitive when it was more of a “derby” fishery, with information gathered via spying more so than active sharing. Pink shrimp fishermen reported that communication often depended on abundance via the crucial influence of processors, who exhibit a buyer’s monopoly. When shrimp are abundant, processors require a consistent rate of shrimp over time, not the maximum amount possible at any given time. To achieve this, they effectively limit the shrimp vessels can land, and determine when that catch can be landed. Consequently, all fishing vessels must remain on dock for often days at a time to ensure they deliver the right weight on the right day. Remaining dockside increases an individual’s need for active and open communication with other fishermen to maintain information on school location. When shrimp are less abundant, processors do not invoke such limits and fishermen do not have to remain on dock. In this case, information on school location is still important, but is more often spread by spying.

Groundfishermen, in contrast, generally were less interested in sharing information, and appeared to us more independent. For example, we heard from several fishermen that they did not rely on other’s information, but instead their individual “business plan”, or strategy for targeting groundfish over the course of the season. They also generally reported that changes in management strategy were not likely to change their approach or encourage communication. Finally, in general, fishermen knowledge about groundfish was deemed as reliable from year to year, as opposed to being variable for a more mobile species such as whiting or shrimp.

These overarching trends were not universal. The interviews also found some variability in fishermen responses, both across and within the fishing fleet. For example, while it was generally indicated that whiting fishermen share more than fishermen targeting the groundfish complex, how much and why they do so was influenced by their age and time in the fishery, their port, and their individual personality and values. Some fishermen also reported caring about their social network, and while fishermen consistently cited the importance of making a living, many also stated they “did not want the fishery to starve”. These responses indicated social networks can themselves be valued, beyond their use in maximizing catch or profit, and the persistence of and some equity across the fleet were also important. The shrimp fishery denoted another outcome beyond catch maximizing: when shrimp are abundant, processor limits made landing a consistent catch over time more important than maximizing landing or profit. In fact, the control of the processors and these limits made maximizing catch impossible. Other motivations were also noted, including the importance of a sustainable fishery and the desire to sell a clean, healthy product to consumers. We also found communities off the water to be important, with fishermen more likely to interact with one another regularly, or having been in the same social circle for a long time, influencing how much they were willing to share information or otherwise communicate.

**Table 1**. Summary of individuals interviewed by state and sector of the fisheries community.

|  | *Fishermen* | *Observer Program* | *Industry Representative* | *Processor* | *Managers* | *Academics* | *NGOs* |
| --- | --- | --- | --- | --- | --- | --- | --- |
| **Oregon** | 13 | 10 | 4 | 2 | 4 | 4 | 0 |
| **California** | 11 | 8 | 3 | 1 | 2 | 2 | 4 |

### Agent-based Model Analysis

To give a better understanding of how the main parameters of the ABM interact to determine resource use, we can compute the expected harvest rate *H* of a typical fishing agent, given ecological and social parameters:

*H* = *C_h_* ∙ *f*

The catchability *C_h_* defines the maximal possible harvest rate given the target (and gear), which would be attained if an agent were to constantly exploit a resource without spending time searching. Thus, we need to compute *f*, the fraction of the season spent in a school, which is the quantity that may be optimized by fishermen behavior, given the ecology of the species they are targeting. It is proportional to a ratio of two important timescales in the system:

*f ~* τ_e,_/τ_s,_

That is, the expected time that an agent can spend exploiting a school, τ_e_, and the expected time it took to find that school, τ*_s_*. Both depend on social behavior: the *cost* of information sharing is that it reduces τ_e_ due to more agents exploiting the same school; the *benefit* is that it also reduces τ*_s_* as agents can exploit their friends’ schools rather than search longer.

The search time τ*_s_* can be computed analytically for our implementation of intermittent search (Barbier and Watson *in review*), using parameters like the radius and number of fish schools, and vessel speed. As for the time spent harvesting, it depends on two factors:

τ*_e_*= min(τ*_l_* , τ*_h_*/*n_o_* ), (1)

where τ*_l_=1/F_p_* is the target species mobility timescale, defined by the rate at which schools move to random locations, τ*_h_* is the expected time for one fishing agent to completely deplete a fish school, and *n_o_* is the expected number of fishing agents on a school at any given time. This final term is complex and incorporates all social effects, but always increases with the probability of information sharing, λ. Hence, τ*_h_*/*n_o_* is the expected time for a school to be entirely depleted. Given *F_s_* the number of fish in a school, and *C* the catchability, we can further define:

τ_h_ = F_s_ / C

These simple equations show how the mobility of the fish (through τ*_l_*) can discount the cost of information sharing λ (which, as noted above, is due to a decrease in τ_e_ the time spent exploiting a school). Indeed, equation (1) shows that, if τ*_l_* is very large (slow-moving schools), τ_e_ will always decrease with *n_o_* and therefore with λ, causing a reduction in catch rate *H*. However, if τ*_l_* is very small (fast-moving schools), τ_e_ = τ*_l_* is not affected by *n_o_*_,_ and hence communication has no adverse effect. In plain words, in the first situation, harvest is limited by the fact that a school is rapidly exhausted, and this is made worse by sharing; in the second situation, harvest is limited by the school moving away, and sharing has no cost.

Qualitatively, the three main factors playing into information sharing are: (1) the harder it is to find a school by oneself, the higher the benefit of sharing; (2) the smaller the schools (the shorter the harvest time), the higher the cost due to competition; and finally (3) fish mobility acts as a discount on that cost, and thus decides whether sharing is beneficial or not.

A full description of the model is already published and available in: Barbier M., & Watson J. R. 2016 The spatial dynamics of predators and the benefits and costs of sharing information. *PLoS Comput. Biol.* **12(10)**, e1005147. (doi:10.1371/journal.pcbi.1005147).
